# Supplementary material for: Effect of Pulsed Electric Fields on the Lipidomic Profile of Lipid Extracted from Hoki Fish Male Gonad
Source: Foods. 2022 Feb 21;11(4):610. doi: 10.3390/foods11040610 (PMC8871228; doi:10.3390/foods11040610)
Supplement: Supplementary file 1 [file foods-11-00610-s001.zip › foods-1576373-supplementary.pdf]

Supplementary Materials

Table S1. Assignment of individual phospholipid spectra in the ^{31}P-NMR analysis of total lipid extracted from HMG.

Phospholipids	Number of phosphorus	Chemical shift (ppm)
PA - phosphatidic acid	1	3.1
LDPG - lyso-diphosphatidylglycerol	1	0.34
CL - cardiolipin	2	0.13
LPSP - lyso-phosphatidylserine-plasmalogen	1	-0.09
SM - sphingomyelin	1	-0.23
PE - phosphatidylethanolamine	1	-0.29
LPC - lyso-phosphatidylcholine	1	-0.38
PS - phosphatidylserine	1	-0.52
PI - phosphatidylinositol	1	-0.72
PC- phosphatidylcholine	1	-0.87

Assignment of individual phospholipid spectra was made relative to PC at -0.87. The 31P-NMR analysis was conducted in a D_2O based detergent system, pH 7.4.

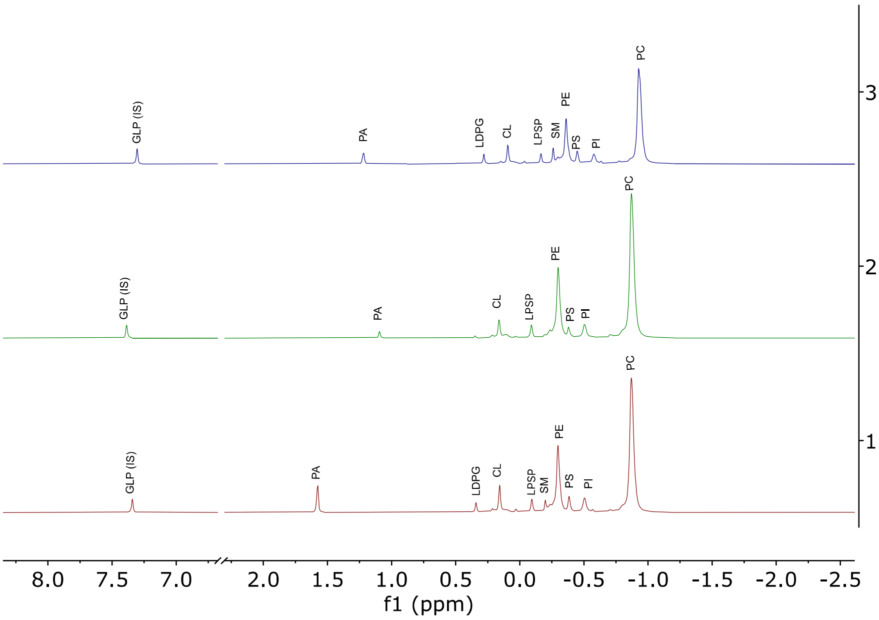

Figure S1. ^{31}P NMR spectra of phospholipid profile for un-heated control, heat-treated control, and PEF treated (50 Hz, 1.25 kV/cm) HMG. 1 = PEF treated, 2 = heat-treated, 3 = un-heated control.
